# Hybrid market design for decentralized energy trading: multi-k double auction with enhanced stable matching

**DOI:** 10.1038/s41598-026-46694-5

**Published:** 2026-05-18

**Authors:** N Reka, S Kuruseelan

**Affiliations:** School of Electrical Engineering, Vellore Institute of Technology, Chennai, Tamil Nadu 600127 India

**Keywords:** Peer-to-Peer (P2P) energy trading, Multi-k double auction, Gale-Shapley matching, Ant colony optimization (ACO), Smart grid, IEEE 37-node test feeder, Prosumer preferences, Energy market efficiency, (ESR) Energy surplus ratio, Electrical and electronic engineering, Photovoltaics

## Abstract

The rise of distributed energy resources (DERs) and sophisticated digital communication systems has led to the emergence of Peer-to-Peer (P2P) energy trading as a viable method for facilitating flexible, autonomous, and efficient energy exchange within Local Energy Communities (LECs). This paper proposes a novel market model for P2P energy trading by integrating a multi-k double auction framework with the Gale-Shapley stable matching algorithm and metaheuristic optimization. The multi-k double auction offers greater pricing flexibility compared to traditional mechanisms such as uniform, discriminatory, and pay-as-bid auctions. The trading price is computed using a weighted parameter k balancing buyer and seller preferences, where k is evaluated for static values (0, 0.5, 1) and optimized dynamically using Ant Colony Optimization (ACO). Buyer-seller matching is performed using a constraint-aware Gale-Shapley algorithm that respects buyers’ budget limits and sellers’ capacity constraints. The model is validated using realistic data derived from photovoltaic generation, load forecasts, and prosumer preferences based on the IEEE 13 and IEEE 37-node residential feeders. Simulations were conducted over hourly trading periods divided into bidding and power exchange intervals. In the present work, the proposed hybrid architecture achieves improved social welfare, fairness in price allocation, higher market liquidity, and enhanced satisfaction for participants. This framework establishes a scalable and adaptive decision-making process that supports stable, efficient, and equitable operations in decentralized energy markets.

## Introduction

The energy sector is undergoing a significant transformation with the rise of renewable energy sources such as solar rooftop panels, Energy Storage (ES) systems, Electric Vehicles (EV), and small wind turbines. This shift is changing the traditional grid into a more dynamic smart grid. Traditionally, electricity was generated at large power plants, transmitted over long distances, and delivered to end-users at a fixed price. In contrast, the smart grid enables electricity to flow in multiple directions, allowing for generation at the distribution level and reducing the need for extensive transmission networks^1^. In this new energy landscape, consumers are evolving into “prosumers,” simultaneously producing and consuming electricity by managing multiple renewable energy sources. This paper explores the implications of this transition, focusing on the challenges and opportunities presented by the integration of renewable energy into the smart grid^2^.A prosumer generates energy using Distributed Energy Resources (DER) at their premises and can sell surplus energy to other consumers or the main grid. This forms a Local Energy Community (LEC), where prosumers and consumers trade energy. However, many distribution networks aren’t designed for reverse power flows from DERs, risking power system stability. Community Energy Storage (CES) offers a solution by allowing energy trading within the LEC at favorable prices, helping maintain system stability and providing economic benefits^3^.

P2P energy trading helps balance supply and demand in the power grid by allowing users to trade electricity directly within their community. Prosumers can sell their excess energy to other consumers at a negotiated price, which is cheaper for consumers and more profitable for prosumers compared to grid tariffs^4^. It reduces the load on the main grid and lowers distribution and transmission losses due to shorter trading distances. P2P trading can also enhances financial benefits for both consumers and prosumers by optimizing energy costs and revenues^5^.

Recent studies in P2P energy trading have predominantly focused on optimizing single parameters like energy demand and pricing schemes, often overlooking coordinated economic benefits for both consumers and prosumers^6,7^. Addressing the gap, a novel framework for local energy communities (LECs) that prioritizes diverse criteria per time interval, such as revenue generation, bill savings, and transaction volume, to determine optimal trading scenarios was introduced^8^.

In contrast to existing auction types, a double auction strategy was used to provide a new market model for P2P energy trading, which gives players more flexibility in negotiating contract prices^9^. The simulation focused on a 1-hour trading period that was split into bidding and power exchange intervals, with special attention to the first 30-minute bidding phase. It employed randomized data from utility load research and photovoltaic owners. The suggested multi-k double auction approach was contrasted with other double auction business models, such as uniform, discriminating, and k double auctions. The results demonstrated that, in contrast to the multi-k double auction method, the pricing mechanisms in these models provide players with little freedom to modify negotiated prices^10–12^. Though the multi-k double auction pricing mechanism has given a highly flexible offer for the participants, still they impose limited flexibility due to the fixation of the equal weighting factor k irrespective of the negotiations of the participants. Hence, it is essential to optimize the weighting factor k based on the buyer and seller notions of behavior. Many optimization methods for peer-to-peer energy networks have been studied, with a particular focus on multilateral commerce that matches customer preferences^13^.One study proposed a two-stage process that resulted in lower customer bills and increased prosumer revenue via optimization and rule-based restrictions^14^. Furthermore, research has explored social collaboration among prosumers for effective energy trading^15^, and another study developed a two-stage optimization technique to promote community engagement^16^.In addition, incentive psychology was employed to induce prosumers to participate in energy trading using a battery storage device^17^.

Game theory is crucial for P2P energy trading, modelling participant competition for financial objectives as a game to find market equilibrium. It includes non-cooperative games, where participants act independently with potential conflicts. Static non-cooperative games involve single actions, while dynamic ones allow multiple actions based on others’ decisions. These games are often solved using Nash equilibrium, ensuring stability where no participant benefits from deviating if others follow equilibrium actions^18^. Machine learning, especially reinforcement learning (RL), has been recently integrated into P2P energy trading, using incentives and penalties to guide actions and improve decision-making over time. RL models aim to converge quickly by reinforcing beneficial actions and penalizing detrimental ones^19^. Current research on optimization algorithms for P2P energy trading faces challenges due to uncertainties like imperfect information and forecast errors. While state-of-the-art methods assume ideal conditions, practical implementations must contend with real-world complexities such as varying prosumer behavior and incomplete data. Integrating machine learning can offer potential solutions by expanding decision-making possibilities, despite posing computational challenges. Future approaches need to blend the strengths of traditional optimization and machine learning to effectively address the specific requirements of P2P energy trading^20^. The goal of this initiative is to provide an adaptive P2P energy trading platform that improves price equity and trade stability inside Local Energy Communities. Current models often depend on fixed pricing strategies and rudimentary matching techniques, neglecting to accommodate changing user preferences, real-time market variations, and stability in buyer-seller relationships. To address this disparity, we present an innovative method that combines a multi-k double auction mechanism with Gale-Shapley stable matching, while also optimizing the price parameter k using Ant Colony Optimization (ACO). This integrated approach guarantees equitable clearing prices, consistent trading alliances, and enhanced societal welfare. The architecture is evaluated using the IEEE 37-node home test feeder, exhibiting enhanced performance for energy exchanged, cost savings, and prosumer satisfaction relative to conventional fixed-k models.

Recent research has extensively explored P2P energy trading, focusing on core areas such as market analysis, business models, and grid operations. Studies have highlighted the potential for growth in P2P markets full of community , hybrid and discussed integrating these markets with existing wholesale and retail frameworks. Challenges identified include scalability issues and the need to address market integration methods to facilitate peer switching between markets for enhanced convenience and efficiency^21^. Furthermore, to address these issues and raise the general dependability and adaptability of P2P energy systems, it has been suggested that advanced technologies like blockchain for transaction transparency, smart contracts for automation, and artificial intelligence for dynamic pricing and demand forecasting be integrated^22,23^.

The proposed system introduces a dynamic multi-k double auction where price flexibility is decided by real-time optimization using ACO, in contrast to previous efforts that either apply fixed auction settings or execute matching exclusively based on static preference lists. Additionally, we include market-based limitations (price, quantity, rating preference and profit preference) into the matching process, in contrast to traditional Gale-Shapley implementations that disregard economic concerns. For hourly P2P trading, this dual-stage optimisation–matching technique offers an adaptive decision-making framework that improves fairness, increases trade efficiency, and creates stable buyer–seller relationships. This work introduces an integrative strategy that brings together multiple complementary components to offer a unique and cohesive approach.

The rest of the paper is organized as follows: Section A systematic P2P energy trading framework gives information about the techniques used in the proposed model. Section Proposed work details the optimized double auction technique. Section Main study energy trading using test feeders presents and discusses the results. Finally, Section Conclusion concludes the paper and offers recommendations for future research.

## A systematic P2P energy trading framework

**Fig. 1:**
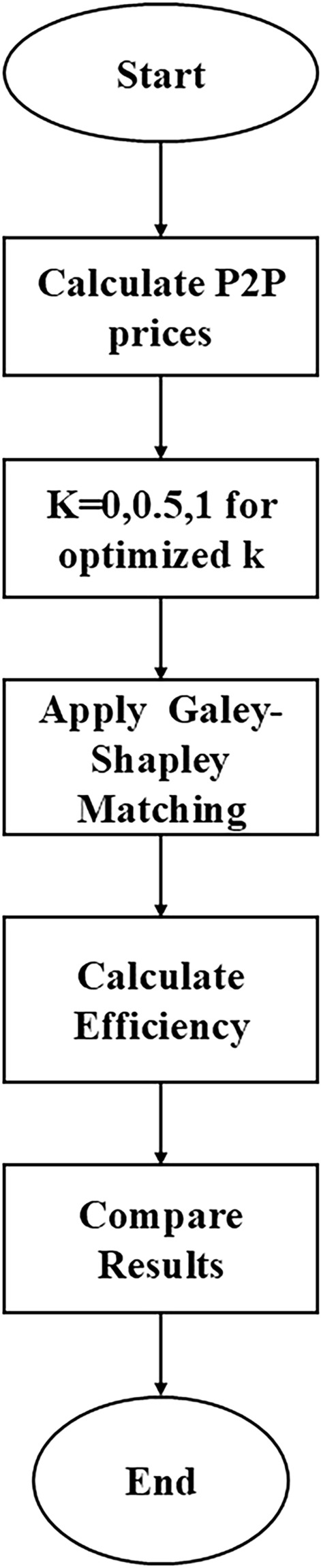
Processes in P2P trading energy-based market.

An efficient framework for facilitating decentralised energy exchange among prosumers while enhancing market efficiency and social welfare is peer-to-peer (P2P) energy trading. P2P markets are typically created by identifying buyers and sellers, gathering bid and offer data, and using market-clearing techniques to ascertain equilibrium prices. Several works have integrated pricing and matching optimisation strategies to improve transaction efficiency and fairness, addressing issues including supply-demand imbalance, participant preferences, and economic incentives. Ant Colony Optimisation (ACO), one of several metaheuristic optimisation techniques, is used to determine the optimal trade parameter k. ACO is highly suited for this challenge due to its powerful global search capabilities and adaptive learning behaviour, allowing for effective convergence towards an optimal P2P clearing price that balances buyer and seller interests. The suggested P2P energy trading platform adheres to a systematic and iterative methodology, as depicted in Fig. 1. The number of buyers and sellers taking part in the market is first determined. After that, important input characteristics are gathered, including energy amounts, bid and offer prices, profit preferences, and rating variables for both buyers and sellers. To enable effective market clearing, seller offers are placed in ascending order while buyer bids are sorted in descending order. The breakeven index is calculated using the sorted bids and offers to determine the equilibrium point where energy supply and demand are satisfied at a reasonable and effective market price. The best market parameter settings are found using the Ant Colony Optimisation (ACO) algorithm, which guarantees a fair trade-off between the interests of buyers and sellers. A matching and pairing procedure is used to match buyers with qualified vendors after the equilibrium price has been determined.

### Comparative analysis of different double auction mechanisms

After establishing the ideal value of k using the Ant Colony Optimisation (ACO) algorithm, the multi-k double auction method is used because it provides a flexible and balanced price structure for peer-to-peer energy trading.. The system adapts efficiently to changing market conditions and heterogeneous consumer preferences by incorporating a tunable parameter (k) that adjusts the relative influence of buyer bids and seller offers. Furthermore, it maintains fundamental economic qualities such as fairness, market efficiency, and incentive compatibility, while allowing for a seamless trade-off between buyer- and seller-oriented pricing. Optimisation of k improves both social welfare and overall market performance, making the multi-k double auction ideal for decentralised P2P energy markets. The physical transfer of energy and payment of the associated transfer fees follow the approval of the transaction in the event of a successful match. The trade process ends for that iteration if there is no viable match, indicating unfavourable market circumstances. Until buyers and suitable sellers are successfully matched, the process is repeated iteratively. This methodology optimises energy allocation and price for all market participants while increasing transaction success rates The structural and operational differences between the studied double auction methods are quantitatively shown in Table 1, which also shows how each model manages market-clearing dynamics, strategic behaviour, and value heterogeneity. Although they have a low computing overhead, traditional processes like uniform and discriminating auctions are unable to internalise participant-specific utility functions, which frequently leads to allocative inefficiencies and less than ideal welfare outcomes. Pay-as-bid auctions reduce price efficiency in high-variability P2P markets because they are more vulnerable to bid shading and dishonest bidding, despite being transparent in settlement. On the other hand, by using weighted price determination, the k-double and multi k-double auction families show a higher level of parametric flexibility, allowing for more precise control over trade-offs between efficiency and justice. However, in order to calculate equilibrium-compatible k values, these methods intrinsically necessitate complicated optimisation, particularly when nonlinear preference structures and stochastic supply-demand situations are included. The proposed ACO-driven multi k-double auction architecture is primarily motivated by the comparative insights from Table 1, which emphasise the need for adaptive, optimization-integrated auction frameworks that can dynamically adjust price parameters and maintain robustness under fluctuating prosumer behaviour.

**Table 1:** Comparative study of Different Double Auction Techniques.

Double auction technique	Mechanism	Advantages	Challenges	References
Uniform Double Auction	All successful trades are executed at a single clearing price determined by intersecting the aggregate demand and supply curves.	Simplifies the trading process, ensures fairness as all transactions occur at the same price.	May not reflect individual valuations of energy, leading to suboptimal outcomes for some participants.	^2–4^
Discriminatory Double Auction	Buyers and sellers pay or receive their respective bid or offer prices. Transactions occur at different prices based on individual bids and offers.	Allows participants to trade at prices they deem fair, reflecting their personal valuations.	Can lead to price discrimination and complex settlement processes.	^8,10,12^
Pay-as-Bid Auction	Buyers pay the price they bid, and sellers receive the price they offer.	Simple to implement, transparent as each participant knows what they will pay or receive.	Can result in inefficiencies if bids and offers vary widely.	^7,18^
k-Double Auction	Uses a weighting factor k to balance the prices paid by buyers and received by sellers. The final transaction price is a weighted average of the bids and offers.	Provides flexibility and can be tuned to balance fairness and efficiency.	Determining the optimal k factor can be complex, requiring sophisticated optimization techniques.	^9–12^
Multi k-double Auction	The factor k can vary, allowing for dynamic adjustment based on market conditions and participant preferences.	Provides greater flexibility and can be tuned to balance fairness and efficiency.	Determining the optimal k factor can be complex, requiring sophisticated optimization techniques and depends on market dynamics.	^4,9^

### Gale-shapley algorithm

The Student-University Matching Problem is a variant of the Gale-Shapley algorithm, used to align students with institutions according to reciprocal preferences. Students rate universities, whereas universities rank students, each with a defined admission capacity. The algorithm works by allowing students to submit proposals to their favorite colleges, who then approve the most favorable proposal according to their rankings while rejecting the rest. Students who were rejected persist in submitting applications to their subsequent preferences. The procedure continues until no more proposal are submitted, guaranteeing a stable match in which no student-university couple would choose to exchange preferences. The outcome may be student-optimal or university-optimal based on the proposer. This method is extensively used in domains such as medical residency matching, educational admissions, and employment placements. The temporal complexity is O(n²), where n is the number of players.

The Gale-Shapley algorithm, or Stable Marriage Problem algorithm, is a technique used to identify a stable matching between two groups of participants with preferences for members of the opposing group. Typically applied to scenarios like matching men and women based on their mutual preferences, the algorithm operates by having each participant propose to their preferred member of the opposite group who hasn’t rejected them yet. Recipients tentatively accept proposals from their most preferred proposers and reject all others. This iterative process continues until no further rejections are possible, resulting in a stable matching where no two participants would prefer each other over their current partners. Mathematically, for sets $B$ (Boy) and $G$ Girl), and individuals $b\in B$ and $g \in G$ the algorithm ensures a stable matching where each pair (b,g satisfies conditions ensuring no instability exists based on individual preferences). The Gale-Shapley approach has a temporal complexity of O(n²), where n represents the number of men or women. In the worst-case scenario, each man proposes to each woman, for a total of two proposals. Making it applicable in a variety of applications. The computational complexity of the proposed framework is primarily determined by the number of participating buyers and sellers in each trading interval. The market-clearing process involves sorting buyer bids and seller offers, which incurs a computational cost of $\mathcal {O}(B \log B + S \log S)$, where *B* and *S* denote the number of buyers and sellers, respectively.

The constraint-aware Gale–Shapley matching algorithm exhibits a worst-case computational complexity of $\mathcal {O}(\max (B,S)^2)$, as each buyer may propose to multiple sellers until a stable matching is achieved. In addition, the eligibility verification and allocation stages introduce linear overhead proportional to the number of feasible buyer–seller pairs.

The computational complexity of the ACO-based optimization of the price parameter *k* increases with the number of ants and the number of iterations. However, since the optimization is performed at the market-clearing level rather than for each individual match, the overall framework remains computationally tractable. Consequently, the proposed architecture exhibits polynomial-time complexity and demonstrates good scalability, making it suitable for real-time and decentralized P2P energy trading implementations.

### Comparison of existing P2P energy trading approaches and the proposed framework

A detailed comparative analysis of existing P2P energy trading approaches and the proposed framework is presented in Table 2

**Table 2:** Comparison of Existing Works and Proposed Approach.

Feature / Criteria	Existing works	Proposed work
Auction Mechanism	Fixed-*k*, Uniform, Discriminatory, or Pay-as-Bid pricing^9,11,12^.	Dynamic Multi-*k* Double Auction with flexible pricing.
Optimization of *k* Static or predefined values (e.g., $k=0$, 0.5, 1)^9,11^.	ACO-based optimization of *k* per-trading interval.	Dynamic k value.
Matching Method	Traditional Gale–Shapley or simple price–quantity matching^7^.	Enhanced constraint-aware Gale-Shapley matching using price, quantity, rating, profit, and capacity.
Preference Consideration	Either price or energy quantity only^6,8^.	Considers both economic (profit) and behavioral (rating) preferences.
Trading Adaptability	Static configuration or parameter-based matching^10,12^.	Hourly adaptive pricing and matching using real-time data.
Market Dynamics Handling	Weak or no integration of supply–demand dynamics^21,22^.	Actively accounts for buyer-to-seller ratio and real-time market variations.
Game-Theoretic Stability	Not ensured or partially discussed^7^.	Stable matching guaranteed with non-blocking pairs.
Metaheuristic Integration	Rarely used for auction pricing, mostly for scheduling^20^.	ACO directly embedded into pricing mechanism.
Dataset Application	Small-scale or simulation-based node models^6,12^.	IEEE 13-node and IEEE 37-node test feeders with realistic solar and load datasets.
Equity & Social Welfare	Primarily focused on pricing or revenue^9^.	Includes social welfare, market liquidity, fairness, and participant satisfaction.
Scalability & Smart Grid Integration	Limited scalability analysis^22^.	Supports decentralized P2P market with smart grid alignment.
Novelty Claim	Optimization , matching and pricing (individually)^6,7^.	Combined adaptive multi-*k* pricing + optimized *k* via ACO + market-based stable matching.

## Proposed work

The proposed work implements a market-based matching mechanism for trading electricity between prosumers (both sellers and buyers) using various parameters like quantity, price, Rating and profit preference. Trading is scheduled from 8:00Hrs to 17:00 Hrs, divided into multiple one-hour rounds, each comprising a 30-minute bidding period and a 30-minute energy exchange period. It involves setting up input parameters like the feed-in tariff (FIT), market price (TOU), a big number (X) for distance calculation, and a premium limit (gamma). The process of the research work is shown in Fig. 4.

### Optimization techniques

#### Ant colony optimization (ACO)

Ant Colony Optimization (ACO) is an optimization method inspired by the foraging behavior of ants. In nature, ants choose the most efficient routes between their colony and food sources by laying down pheromones-chemical signals that guide other ants along optimal paths. ACO utilizes this behavior to address intricate optimization challenges by simulating the pheromone deposition and route selection mechanisms of ants^24,25^.

The algorithm iteratively improves candidate solutions by balancing exploration (trying new paths) and exploitation (reinforcing good paths), which makes it particularly effective in handling problems with numerous possible solutions.

**Fig. 2:**
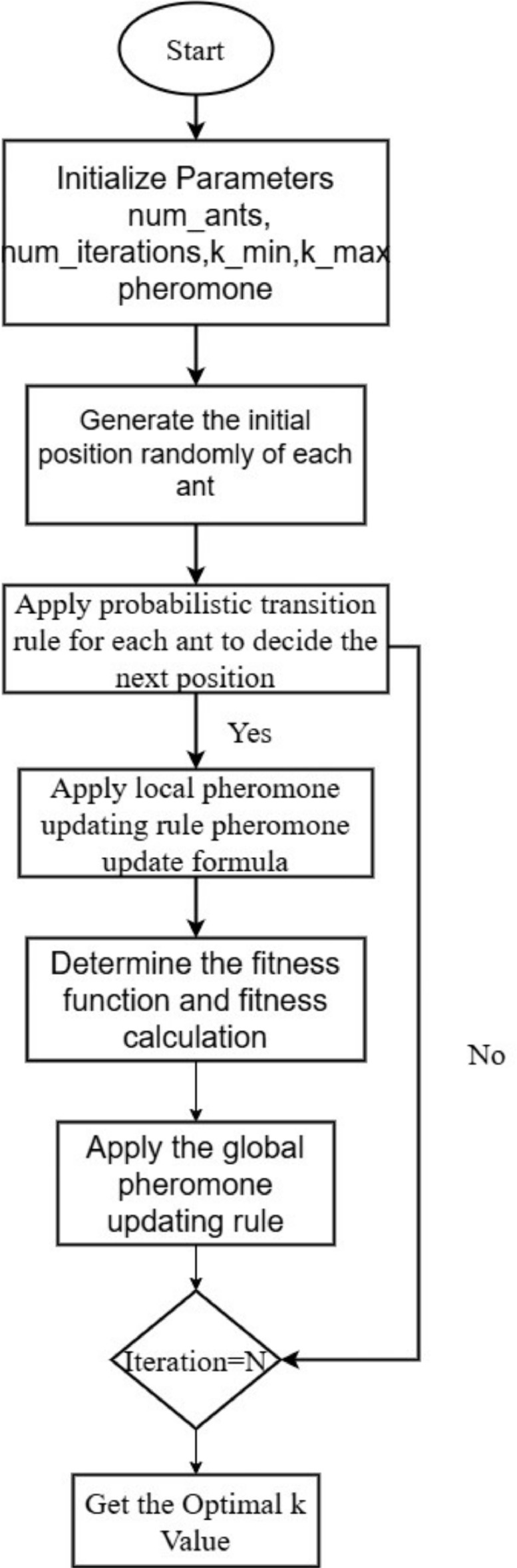
Flowchart for Ant Colony Optimization.

ACO is a probabilistic technique derived from the foraging behavior of ants, aimed at addressing complex optimization problems^26,27^ . In this approach, artificial “ants” construct solutions by traversing a problem space, influenced by pheromone trails that represent the quality of previously found solutions. These pheromones are updated dynamically to reflect the success of routes taken, guiding subsequent ants toward increasingly optimal solutions (Fig. 2).

**Fig. 3:**
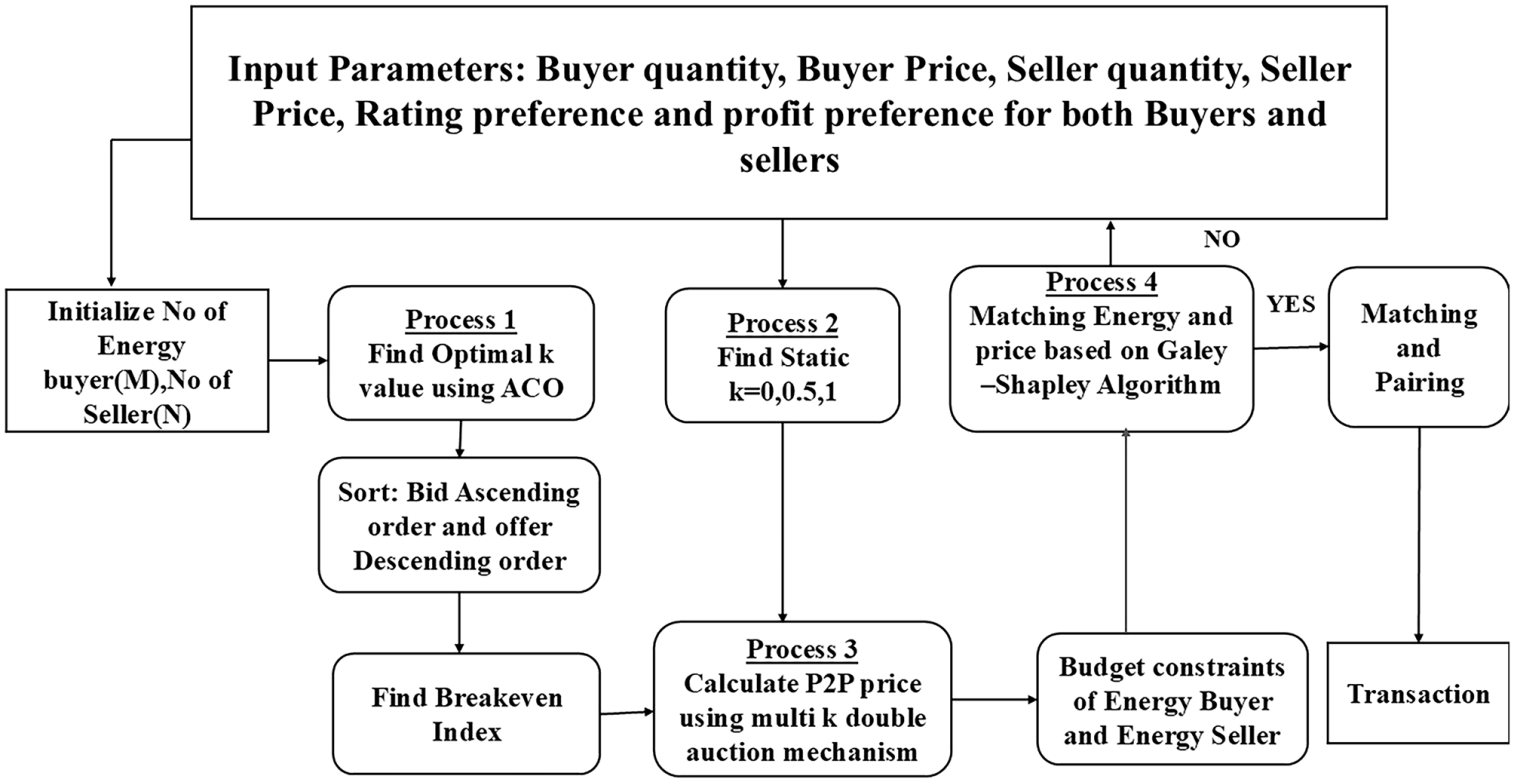
The process of Research Methodology.

Figure 3 illustrates the flowchart of the ACO algorithm.

1 $$\begin{aligned} P_{ij}^{k}(t) = {\left\{ \begin{array}{ll} \dfrac{[\tau _{ij}(t)]^{\alpha } [\eta _{ij}]^{\beta }}{\sum \limits _{l \in \mathcal {N}_i^k} [\tau _{il}(t)]^{\alpha } [\eta _{il}]^{\beta }}, & \text {if } j \in \mathcal {N}_i^k \\ 0, & \text {otherwise} \end{array}\right. } \end{aligned}$$

where $\tau _{ij}(t)$ is the pheromone concentration on the path from node $i$ to node $j$ at time $t$, $\eta _{ij}$ is the heuristic value (typically inverse of distance or cost), $\alpha$ and $\beta$ are control parameters that regulate the influence of pheromone and heuristic information respectively, and $\mathcal {N}_i^k$ is the set of feasible nodes that ant $k$ can visit from node $i$.

The pheromone levels are then updated based on the quality of the solutions found. The general pheromone update rule is given by:

2 $$\begin{aligned} \tau _{ij}(t+1) = (1 - \rho ) \cdot \tau _{ij}(t) + \Delta \tau _{ij}(t) \end{aligned}$$

where $\rho \in [0,1]$ is the pheromone evaporation rate, and $\Delta \tau _{ij}(t)$ represents the amount of pheromone deposited, usually proportional to the quality of the solution traversing edge $(i, j)$.

To avoid premature convergence to suboptimal solutions, pheromone intensity is reduced over time. This process is represented by:

3 $$\begin{aligned} \tau _{ij}(t+1) = (1 - \rho ) \cdot \tau _{ij}(t) \end{aligned}$$

#### ACO implementation for k-value optimization

The specific optimization task in this study is to determine the value of k that maximizes overall market welfare while maintaining fairness. Every ant is treated by ACO as a potential k value in the interval [0,1]. The colony is directed toward an ideal area by pheromones that are reinforced around k values that result in improved performance. This enables the system to manage the auction-matching process’s nonlinear, preference-driven character, which is challenging to represent in linear or MILP form.

This design ensures fairness, high market liquidity, and stable trading outcomes for the proposed decentralized energy market.

#### ACO based multi-k double auction technique with market based Gale Shapley matching

The specifics of the parameters and variables pertinent to the proposed work are shown in Table 3.The algorithm starts with sellers and iterates through the sorted buyer’s utilities. If a seller can provide the requested power, a match is made, and the quantities are adjusted to prevent further matches for the involved seller and buyer. Similarly, buyers are matched with sellers based on their constraint. After the market closes, the total revenue and expenses for all participants for one day are summarized and presented using bar graphs to compare the performance and flexibility of the proposed method.

#### Analysis of ACO parameter sensitivity, operational viability, and algorithmic complexity

The suggested ACO-based multi-k double auction with stable matching improves trade performance, real-time viability and computational burden must be taken into account for its practical implementation. The complexity of the Gale-Shapley method is O(n²), whereas ACO iterations increase with colony size and participant count. As the number of prosumers increases, repeated bidding rounds and pheromone updates may induce latency in real-time decision-making. Furthermore, communication latency, data quality, and dynamic behavioural changes may influence system responsiveness in field deployment. These difficulties suggest that in order to scale the framework to large communities, distributed computation, lightweight heuristics, or hybrid ACO,Multi-k double auction and Gale-Shapley matching prediction models may be required.

The number of ants, pheromone evaporation rate ($\rho$), and heuristic weighting factors ($\alpha$ and $\beta$) all have an impact on how well ACO optimises the weighting factor *k*. Preliminary sensitivity testing indicates that excessively low evaporation rates can impede convergence, while very high values may cause unstable or early convergence. Similarly, increasing the number of ants boosts exploration but raises computational cost. The robustness of the optimisation procedure was demonstrated by the consistent *k* values achieved by balanced parameter settings over several runs.

**Table 3:** ACO Algorithm Parameters and Variables for Energy Trading.

Category	Parameter	Description
ACO Parameters	num_ants	The number of ants employed in the ACO algorithm. Each ant represents a possible trading strategy or solution (k value) within the energy trading market.
	num_iterations	The number of iterations the ACO algorithm will run. More iterations enhance the likelihood of finding the best answer.
	evaporation_rate	Refers to the rate at which pheromone trails dissipate. It maintains a balance between exploring new solutions and reinforcing good ones.
	k_min / k_max	The range of the *k* value, used to determine trading price via: $P^* = k_i \cdot p^b_i + (1 - k_i) \cdot p^o_i$.
	rating_preference	A parameter affecting the fitness function by giving preference to higher-rated traders.
	profit_preference	A parameter that encourages matches yielding higher profits.
ACO-specific Variables	pheromone_array	Represents each solution’s pheromone strength. Higher levels indicate more promising solutions.
	best_solution	The best *k* value found across all iterations.
	best_fitness	Fitness score associated with the best *k* value.
Sorting & Matching	buyer_offer_price_sorted	Sorted list of buyer bid prices.
	seller_offer_price_sorted	Sorted list of seller offer prices.
	bid index / offer index	Index mappings to retrieve original price and quantity data.
Breakeven & Equilibrium	breakeven index	The point where a buyer’s bid equals or exceeds a seller’s offer.
	p2p_price	Final trade price computed using *k* and breakeven data.
Gale-Shapley Variables	buyer matched / seller matched	Boolean arrays indicating matched participants.
	buyer preferences	Array of buyer rankings of sellers based on price and quantity.
	trading prices	Calculated prices for each buyer-seller pairing.
	buyer quantities / seller quantities	Quantity arrays for matched trades.
Utility & Matching Matrices	utility matrix	Measures utility for each potential match based on price and quantity differences.
	match matrix	Binary matrix showing actual buyer-seller matches (e.g., in a spy plot).

### Multi k-double auction technique

**Fig. 4:**
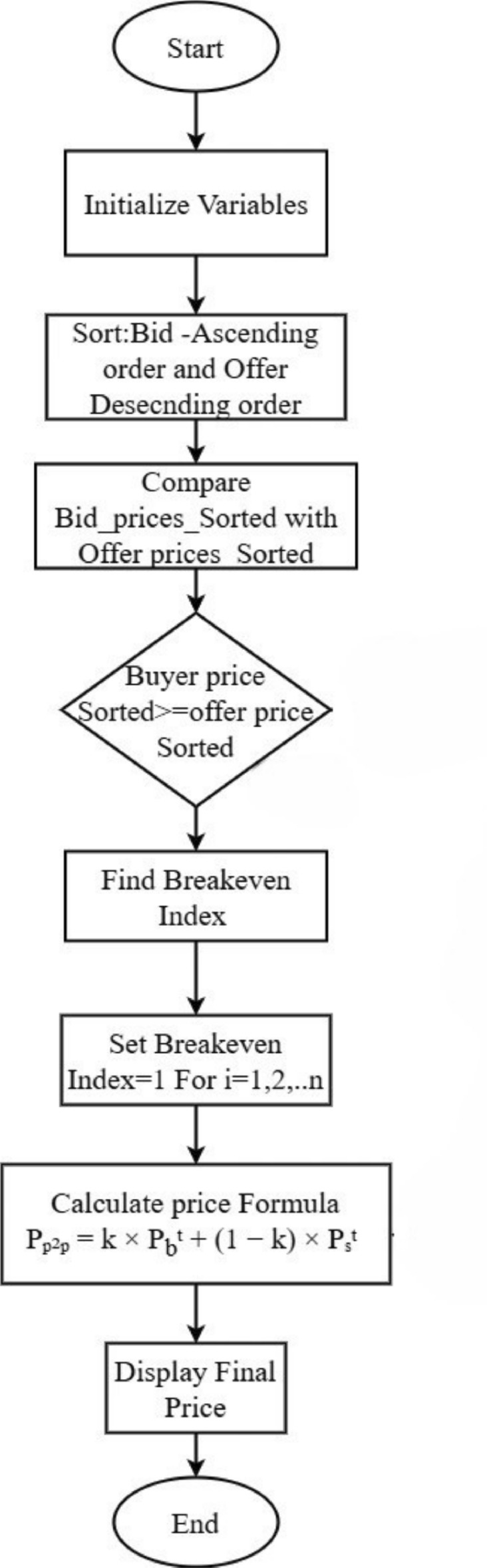
Multi k-double auction mechanism.

The P2P energy trading involves a structured process where prosumers with solar PV rooftop systems and consumers participate. Prosumers must estimate both photovoltaic (PV) generation and load consumption for each time interval, whereas consumers just need to forecast load consumption. Prosumers who have excess energy after meeting their own consumption needs act as energy sellers, while those who generate less than or equal to their use, along with consumers, act as energy buyers. During the bidding process, sellers submit offers with the minimum price they are willing to sell (in ₹/kWh) and the quantity of power (in kW) they are willing to sell, while buyers submit bids with the maximum price they are willing to buy (in ₹/kWh) and the quantity of power they are willing to consume. The exchange price for each buyer-seller pair is determined using a weighting factor k using equation (1). The flowchart of the Multi k- Double Auction mechanism is shown in Figs. 2, 4.

4 $$\begin{aligned} P_{\textrm{P2P}}^{i,t} = k \cdot P_{\textrm{b}}^{t} + (1 - k) \cdot P_{\textrm{s}}^{t} \end{aligned}$$

Let $P_{\textrm{P2P}}^{i,t}$ denote the peer-to-peer (P2P) trading price for pair $i$ at time $t$. Let $P_{\textrm{s}}^{t}$ and $P_{\textrm{b}}^{t}$ represent the recommended prices of the seller and buyer, respectively, for the same pair and time. The designated weighted component, denoted as $k$, signifies the negotiated equilibrium between the price preferences of the buyer and supplier. The resultant P2P trading price is calculated as a convex combination of the buyer and seller prices.

### Market based Gale Shapley matching

Extending beyond Gale-Shapley to incorporate these market-oriented considerations involves designing algorithms that not only take preferences into account but also integrate economic factors like prices, utilities, and various constraints. This approach aligns more closely with market design principles, ensuring that the resulting matches are not only stable but also efficient and aligned with broader economic objectives. The Gale-Shapley algorithm, also known as the Deferred Acceptance algorithm, is a classical method for solving the stable matching problem based on preferences alone. It ensures that both sides of a matching get the best feasible outcome according to their stated preferences. These additional considerations are crucial in market-oriented settings because they reflect economic realities and the interactions between supply and demand. In the proposed work prosumers with equal generation and consumption are excluded from participation.

#### Proposed matching algorithm

Mathematical Representation of Gale-Shapley Algorithm (Buyer-Seller Matching)

- **Sets and Definitions**
  $B = \{B_1, B_2, \ldots , B_m\}$: Set of buyers.
  $S = \{S_1, S_2, \ldots , S_n\}$: Set of sellers.
  $q_j$: Capacity of seller $S_j$, where $j \in \{1, 2, \ldots , n\}$.
  $P_B(B_i) = [S_{j_1}, S_{j_2}, \ldots , S_{j_n}]$: Preference list of buyer $B_i$, sorted by most preferred seller.
  $P_S(S_j) = [B_{i_1}, B_{i_2}, \ldots , B_{i_m}]$: Preference list of seller $S_j$, sorted by most preferred buyer.
- **Matching Representation**
$$M = \{(B_i, S_j) \mid B_i \text { is matched to } S_j\}$$
  Constraint: $|M(S_j)| \le q_j$ for all *j*, ensuring each seller does not exceed its capacity.
- **Stability Conditions**
  1. Individual Rationality
    A buyer $B_i$ is matched to a seller $S_j$ only if they both prefer each other over being unmatched:
    $$\begin{aligned} (B_i, S_j) \in M \Rightarrow S_j \in P_B(B_i) \text { and } B_i \in P_S(S_j) \end{aligned}$$
  2. No Blocking Pair
    For any unmatched pair $(B_i, S_j)$:
    $$\begin{aligned} S_j \in P_B(B_i) \text { and } B_i \in P_S(S_j) \end{aligned}$$
    And if $S_j$ is already matched to $B_k$ (least preferred among matched buyers), then:
    $$\begin{aligned} P_S(S_j, B_i) < P_S(S_j, B_k) \end{aligned}$$
- **Mathematical Optimization form**
  1. Objective Function
    Maximize the total preference satisfaction for all buyers and sellers:
    $$\begin{aligned} \max \sum _{(B_i, S_j) \in M} \left[ P_B(B_i, S_j) + P_S(S_j, B_i) \right] \end{aligned}$$
  2. Constraints
    2.1. Capacity Constraint:
    $$\begin{aligned} \sum _{(B_i, S_j) \in M} 1 \le q_j, \quad \forall j \end{aligned}$$
  3. Stability constraint:
    If a pair $(B_i, S_j)$ is not in *M*:
    $$\begin{aligned} (B_i, S_j) \notin M \Rightarrow P_B(B_i, S_j)< P_B(B_i, M(B_i)) \text { or } P_S(S_j, B_i) < P_S(S_j, M(S_j)) \end{aligned}$$
    The Gale-Shapley algorithm begins by initializing all buyers and sellers are unmatched. The process then considers all the constraints, including the preferences of buyers and sellers. Each buyer proposes to the seller on their preference list. Sellers tentatively accept the highest-priced offers within their capacity and reject the rest. Prices are then adjusted to reflect the market equilibrium, and utilities for the remaining unmatched buyers are calculated. These unmatched buyers continue proposing to the next seller on their list, considering the adjusted prices. Sellers, again, tentatively accept the highest-priced offers without exceeding their capacity. This process of proposal, acceptance, and adjustment is repeated until no further proposals can be made, and all constraints are satisfied.

## Main study energy trading using test feeders

### Simulation study with IEEE 13 Node test feeder

The study focuses on P2P energy trading among prosumers with solar PV rooftops and consumers. Trading occurs from 8:00 Hrs to 17:00 Hrs during peak solar hours, divided into nine rounds of bidding and energy exchange periods. Prosumers predict PV generation and consumption; sellers offer excess energy, while buyers seek energy deficit solutions. The dataset includes six prosumers as sellers and six consumers, detailing PV generation, demand types, preferences, and trading parameters for optimizing P2P energy distribution.

**Fig. 5:**
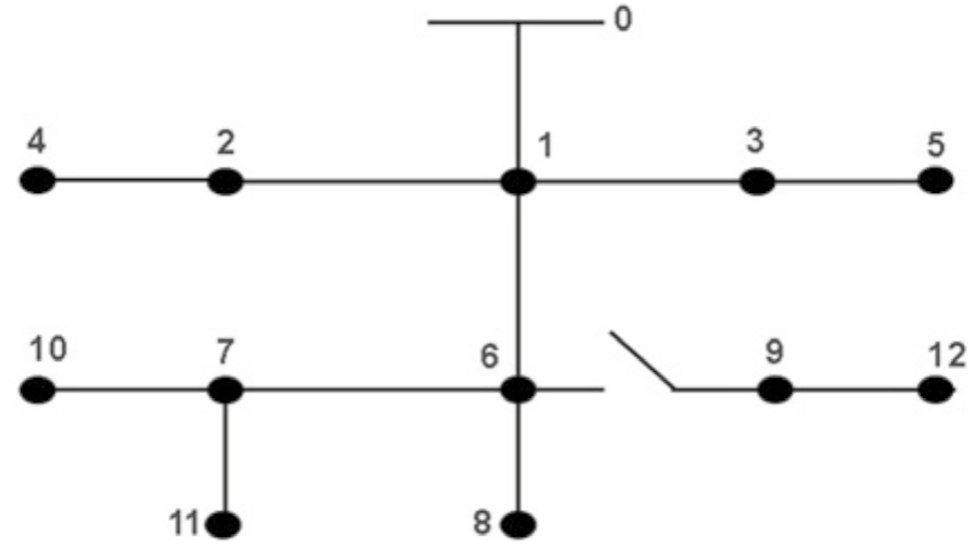
The IEEE 13-bus distribution test system.

The congestion problem is not considered when studying the matching process. Figure 5 depicts a modified test feeder with six sellers (nodes 3, 5, 6, 8, 10, and 13) and six purchasers (nodes 2, 4, 7, 9, 11, and 12) with power quantity and price. Matching buyers and sellers in the IEEE 13 Node Test Feeder for energy trading involves maximizing energy exchange based on preferences such as price, quantity, rating, and profit. The process starts by collecting data on each node’s energy usage and production, followed by specifying these preferences. The goal is to enhance buyer satisfaction while minimizing selling costs. Pair sellers and buyers based on preferences to match supply and demand at the clearing price where they intersect.

### Off peak hours (09:00 Hrs to 10:00 Hrs)

**Fig. 6:**
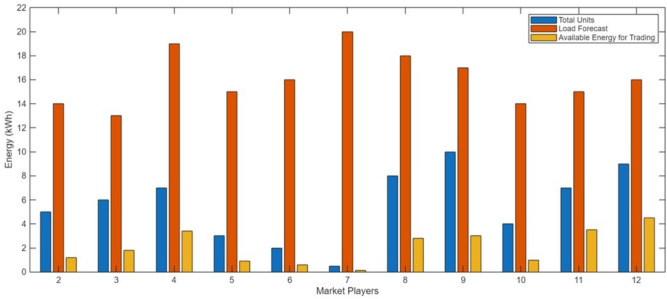
Available Energy for Market Transactions.

The process of matching with 12 participants and segregating them into sellers and buyers with the energy available and the load requirement of the prosumer is performed for one hour of a day, from 09:00 Hrs to 10:00 Hrs which is an off-peak hour. Figure 6 presents a detailed analysis of energy consumption and production across twelve prosumers. Each prosumer’s energy usage is compared with their load forecast to determine the available energy, which represents the surplus or deficit they have. Prosumers with a positive available energy, such as Prosumers 3, 5, 6, 8, and 9, have a surplus, meaning they produce more energy than they consume and can supply this excess to the buyer. Conversely, consumers 2, 4, 6, 7, 9, and 10 face deficits, where their energy consumption exceeds their production, requiring them to obtain additional energy, hence serving as buyers. Notably, Prosumers 1 and 10 have balanced their energy usage perfectly with their forecast, leaving no surplus or deficit, making them neutral in energy trading

**Table 4:** Buyer–Seller Matching Over a One-Hour Interval.

Buyer ID	Buyer price (₹/kWh)	Seller ID	Seller price (₹/kWh)	Traded quantity (Units)	Trading price
2	8	5	9	0.424	8.0015
4	10	8	9	0.862	8.0005
6	9	3	9	0.672	9.0000
7	7	9	8	0.414	7.9995
10	9	12	7	0.559	8.9990

Table 4 shows the sample of matching done for a set of five buyers and five sellers. The Table 4 details the trading transactions between buyers and sellers, including prices and quantities traded. Buyer 2 purchased 0.424 units from Seller 5 at a seller price of 8.0015 ₹/kWh, resulting in a trading price of 8.0015 ₹/kWh. Similarly, Buyer 4 bought 0.862 units from Seller 8 at 9 ₹/kWh, with a trading price of 8.0005 ₹/kWh. The table illustrates the price adjustments and quantity traded in each transaction, reflecting the negotiated trading prices based on buyer and seller preferences. The matching of sellers and buyers is processed with the proposed market-based Gale-Shapley matching and presented in Table 4.

**Table 5:** Buyer–Seller Mapping for a Full-Day Trading Window.

Time (Hr)	Matched (Buyer Id, Seller Id)	Traded Quantity (kWh)	Trading Price (₹)
7th	(12, 5), (11, 3)	0.14375, 0.4245	8.13
8th	(2, 6), (3, 9)	0.05, 0.1025	8.5
9th	(2, 5), (3, 8), (11, 9)	0.0455, 0.315, 0.1165	8
10th	(2, 6), (3, 9)	0.05, 0.1025	8.5
11th	(2, 3)	0.095	7.5
12th	(5, 2), (8, 3), (9, 11)	0.0925, 0.16, 0.065	8
13th	(5, 2), (8, 3), (9, 11)	0.0925, 0.16, 0.065	7.5
14th	(8, 2), (12, 5), (11, 3)	0.14375, 0.4525, 0.2025	8
15th	(2, 5), (3, 8), (9, 11)	0.0455, 0.315, 0.1165	7
16th	(5, 2), (8, 3), (9, 11)	0.0925, 0.16, 0.065	8.5
17th	(2, 11)	0.0725	7

Table 5 shows the energy market for prosumers is intricate, characterized by both surplus and deficit scenarios as buyers and sellers engage to equilibrate energy demands. The market’s clearing efficiency varies every hour, reflecting differing degrees of effectiveness in aligning supply with demand.

Hour 7 has a poor efficiency of 3.73%, with hardly a small portion of extra energy being exchanged. Hour 8 has a notable decline to 7.37%, with little energy transferred despite several vendors providing excess supply. In Hour 9, efficiency improves marginally to 17.38%; nonetheless, a significant portion of the extra energy remains untraded.

During Hour 10, the efficiency remains low at 7.37%, with very few transactions taking place. Hour 11 demonstrates a slight increase in efficiency to 7.65%, however, energy matches remain constrained. Hour 12 shows a similar situation to Hour 11, with trades constituting only a small fraction of the extra energy. The market’s performance remains subpar in Hour 13, exhibiting little energy exchange.

Hour 14 signifies a pivotal moment, exhibiting a substantial rise in efficiency to 23.73%. Significant energy exchanges occur, signifying improved alignment of supply and demand. In Hour 15, efficiency declines to 17.38%, and extra energy remains under utilized. Both Hour 16 and Hour 17 exhibit poor efficiency (7.24%), characterized by a negligible number of successful transactions between buyers and sellers. The market continues to encounter difficulties in maximizing excess energy exchanges, resulting in many prospective deals staying unfulfilled. Enhancing the matching algorithms and pricing techniques may improve efficiency and facilitate more energy trading.

Table 6 presents the results of a one-hour from 12:00-1:00 PM peer-to-peer (P2P) energy trading simulation using the Ant Colony Optimization (ACO) method. The optimal k value determined is 0.9857, indicating that the transaction price is mostly affected by the seller’s proposition. The ideal fitness value of 0.34167 signifies the effectiveness of the selected solution in achieving a balance between fairness and efficiency according to the goal function. The breakeven index is 2, presumably indicating the buyer-seller combination with the minimal profit differential. The ultimate P2P price determined with the optimal k is ₹8.2857. This pricing guarantees a mutually advantageous transaction for both buyers and sellers. ACO efficiently identifies the best parameters within specified limitations. The outcome substantiates the viability of using ACO for dynamic energy pricing inside smart grid systems.

**Table 6:** Metric Values Over One Hour(12:00-01:00 PM).

Metric parameters	Metric value (ACO)
Best K Value	0.9857
Best Fitness	0.34167
Breakeven Index	2
P2P Price (₹)	8.2857

### Simulation study with IEEE 37 node test feeder

**Fig. 7:**
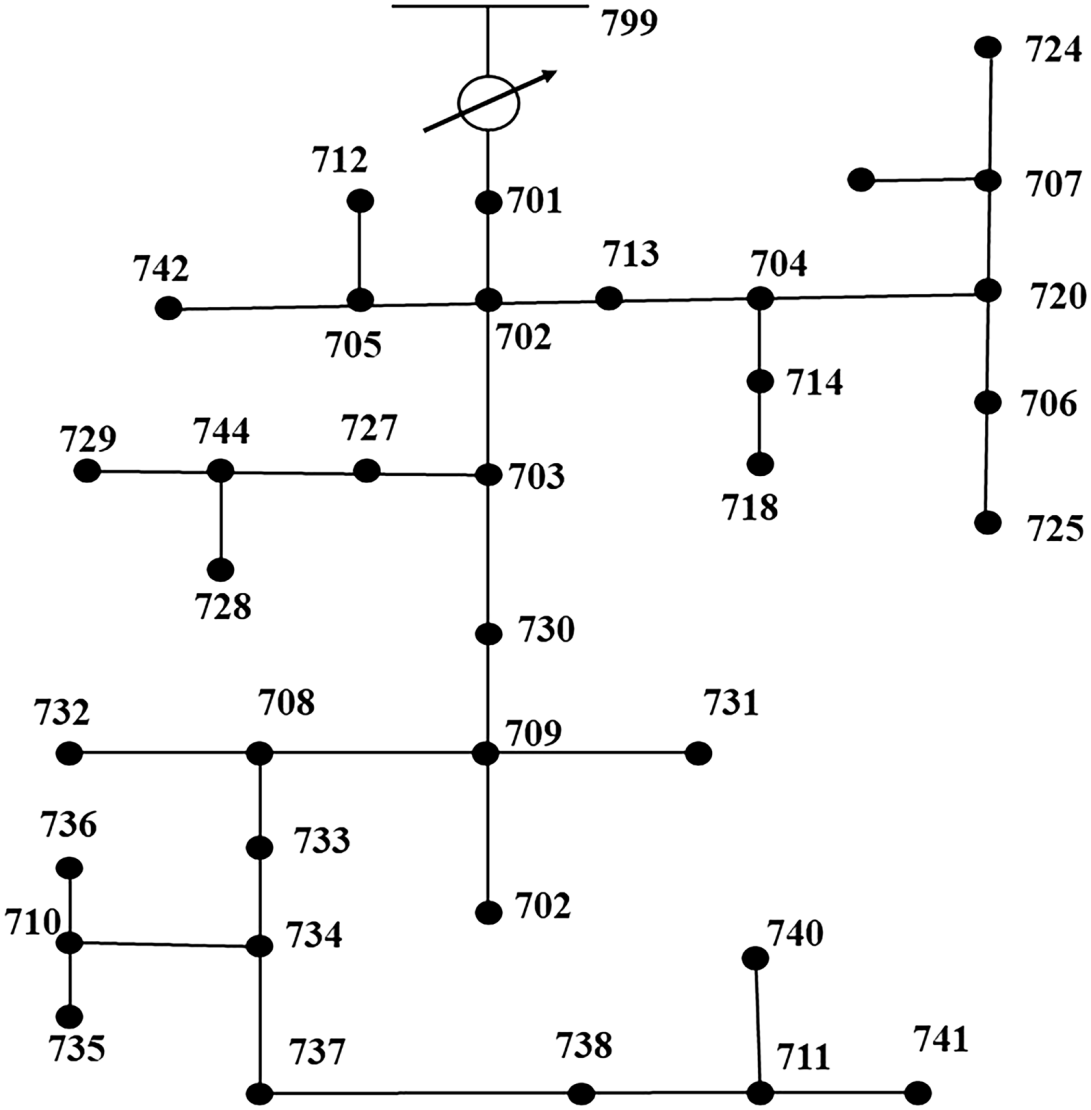
The standard IEEE 37-node feeder model.

The IEEE 37-node test feeder, depicted in Fig. 7, exemplifies a conventional radial distribution network frequently employed for study and simulation in power system analysis. The system comprises 37 nodes (buses) arranged in a single-feeder layout, with multiple lateral branches branching from the main trunk. The substation, or feeder head, is situated at node 799, from which power is disseminated across the network. This testing system is defined by unbalanced loading and single-phase laterals, rendering it suitable for assessing the behavior of real-world distribution systems under diverse scenarios. It is extensively utilized for analyzing power flow, fault detection, voltage management, and the integration of distributed energy resources (DERs) in smart grid applications. The configuration exemplifies standard urban or suburban feeder designs and acts as a dependable reference for evaluating algorithms and control methodologies. This section summarizes the findings of simulation tests evaluating the performance of the proposed model for energy trading in a prosumer-based community. Every prosumer has both flexible and inflexible demand. This study assumes that $\Delta t$ is equal to one hour. The solar production power and electricity consumption of prosumers may be accessed via an online supplement from^28^. This section offers experimental findings of a suggested energy trading strategy in a P2P market.

The goal is to maximize green energy use from DERs while protecting purchasers’ data privacy and maximizing societal welfare. Simulations of 22 P2P market participants over one year use data from^29^. Daily time is split into 48 equal 30-minute intervals. To address energy demand-supply imbalances, the utility grid is used. In the P2P market, buyers want to purchase energy, while sellers seek to sell it. Prices for buyers and sellers are dependent on the SDR. To maximize green energy consumption, customers are classed accordingly^29^. When $ESR \le 1$, energy demand exceeds available surplus, indicating a peak period. According to demand, purchasers are categorized as cooperative (demand less than peak factor) or non-cooperative (demand more than peak factor) in increasing order^30^. Considering the peak factor, purchasers who contribute to peak periods pay peak prices. The proposed scheme’s efficacy is assessed by comparing energy costs and profits (energy bill and income) for buyers and sellers to the grid and a prior research^31,32^. The study suggests a real-time P2P energy trading paradigm for the DR program. Additionally, it functions on a first-come, first-served basis without competition among participants. If a high-demand buyer comes first at a peak, they may acquire all available energy from the P2P market, forcing other purchasers to pay higher grid pricing. Additionally, data privacy is not taken into account. Due to greater expenses and maintenance, we did not include an Energy Storage System (ESS) in our analysis^33^.

In a smart grid environment, it is vital to relay purchasers’ energy demand data to the utility system. To overcome this, the results section is broken into four sections. Evaluation comes first. When contemplating usage-based charging for P2P purchasers, consider the SDR ratio and dynamic peak hours^34^. We compare the findings to the grid and the suggested research in . Second, the suggested strategy is tested and compared to research for consumer satisfaction. Thirdly, private data is sent to the utility grid, including differential noise ratings (Differential Data Reporting). Finally, we analyze the suggested mechanism’s environmental and economic implications^35^.

**Fig. 8:**
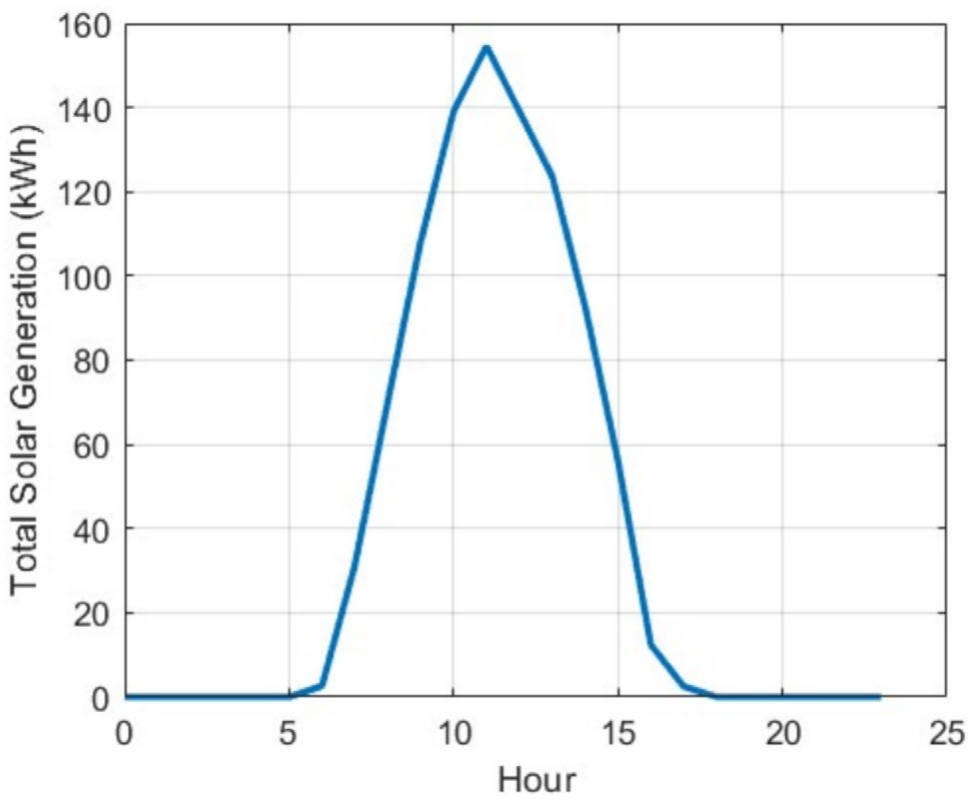
The 24-hour solar generation profile in kWh.

For a 24-hour period, the Fig. 8 shows the diurnal profile of total solar energy output in kWh. The highest production of 155 kWh occurs during noon (about 11:00–12:00) after solar output begins at 06:00. After this high, production slowly drops to insignificant levels by 18:00. This generation profile matches the normal solar irradiance curve, highlighting photovoltaic systems’ operating window for grid-level energy management and peer-to-peer trading.

**Fig. 9:**
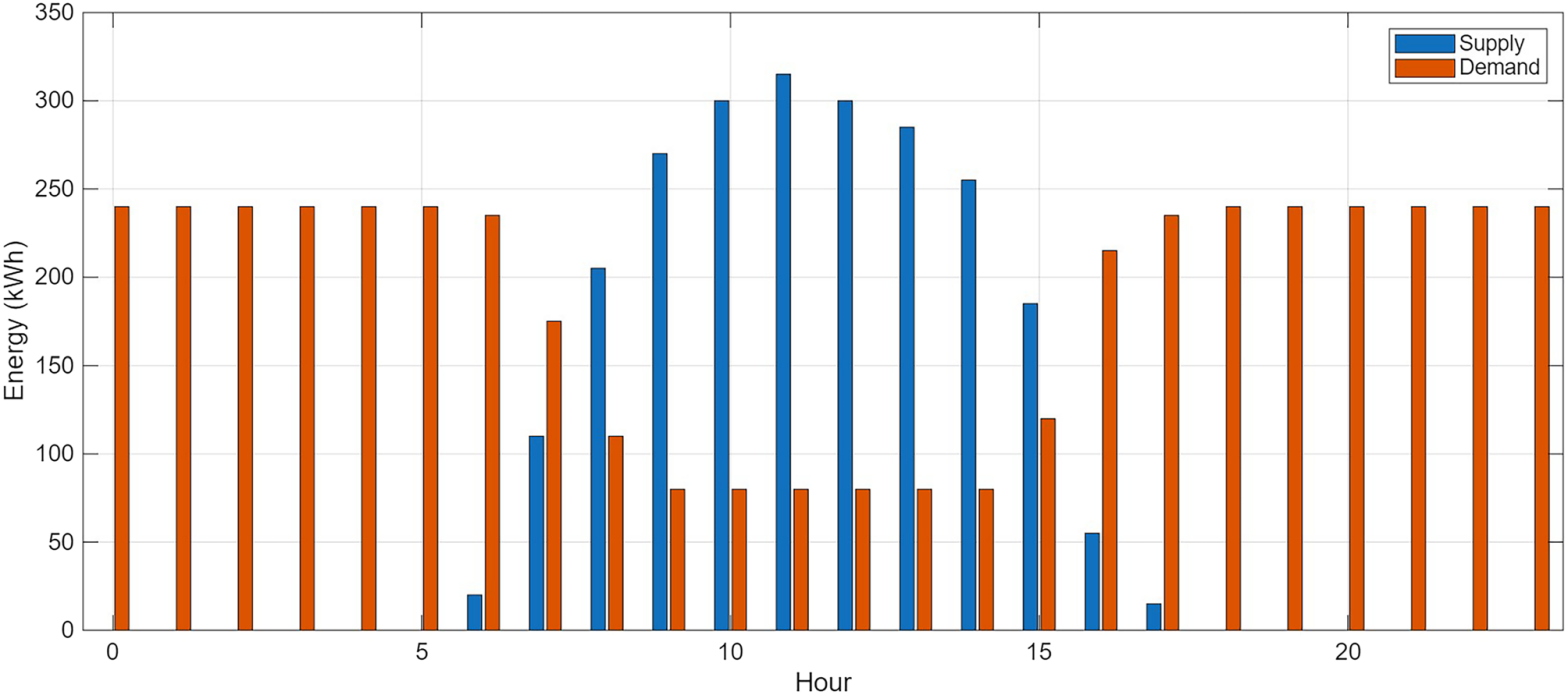
The hourly variation of energy supply and demand over a 24-hour period.

Figure 9 illustrates the hourly variation of energy supply and demand within a 24-hour period. The demand profile remains nearly constant across all hours at approximately 240–245 kWh, indicative of a stable load requirement. In contrast, the supply profile exhibits a strong diurnal pattern consistent with solar photovoltaic generation. Energy supply commences around 06:00, peaks near midday with a maximum output exceeding 300 kWh, and diminishes to zero by 18:00. During daylight hours, particularly between 09:00 and 15:00, the supply surpasses demand, whereas at night and in the early morning, the supply is insufficient to meet demand. This imbalance emphasizes the critical role of energy storage systems and grid-based backup to maintain reliability and continuous energy availability in solar-integrated peer-to-peer (P2P) energy trading frameworks.

**Fig. 10:**
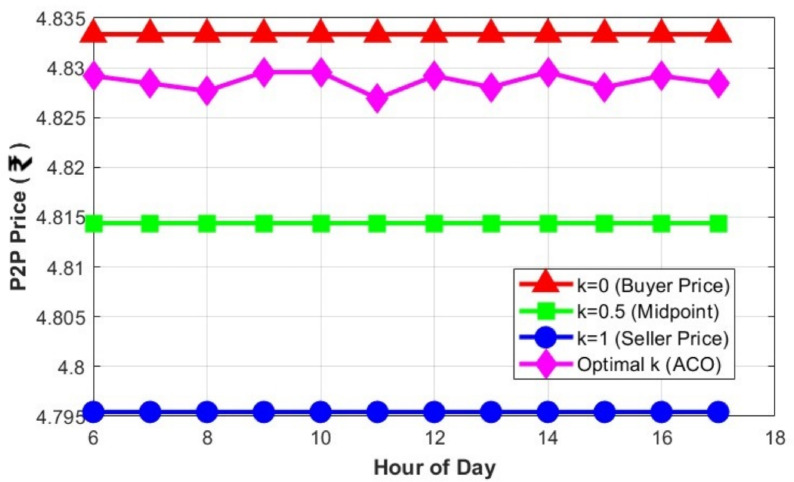
The daily variation in Peer-to-Peer (P2P) energy prices.

The Fig. 10 depicts the fluctuation of Peer-to-Peer (P2P) energy prices over the course of the day, using various pricing schemes influenced by the parameter k, which dictates the weighting of buyer and seller preferences. The buyer-based price (k = 0) is the highest, indicating the maximum amount purchasers are prepared to pay, while the seller-based price (k = 1) is the lowest, denoting the minimum acceptable offer from sellers. The midpoint price (k = 0.5) provides a constant average between the two values. The ideal pricing, determined by an Ant Colony Optimization (ACO) algorithm, fluctuates somewhat over time to maintain market equity and efficiency. This technique guarantees that neither buyers nor sellers are unduly advantaged, making it an appropriate strategy for dynamic and fair pricing in peer-to-peer energy trading systems.

**Fig. 11:**
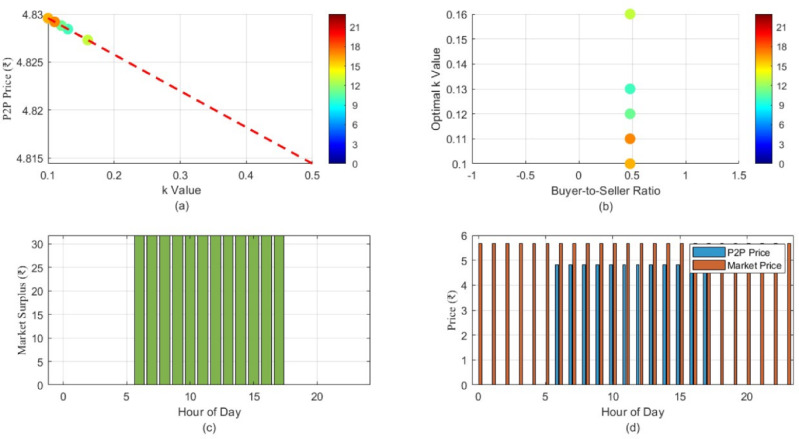
Analysis of P2P pricing trends within the energy market.

This composite Fig. 11 provides a comprehensive investigation of P2P price patterns in an energy market via four subplots. Subplot (a) illustrates the negative correlation between the P2P price and the k value, indicating that elevated k values, which favor sellers, lead to decreased P2P pricing. The color bar represents various hours of the day, illustrating the temporal variations in ideal price. Subplot (b) illustrates the relationship between the ideal k value and the buyer-to-seller ratio, indicating that when the ratio fluctuates, the optimal k adjusts to sustain market equilibrium, highlighting the need for dynamic pricing contingent upon market composition. Subplot (c) depicts the market surplus at various hours, which constantly stays up, signifying effective and advantageous trading results under the optimized pricing system. Finally, subplot (d) just exposes the P2P pricing with the traditional market price throughout the day, demonstrating that the P2P price constantly stays lower, highlighting the economic benefit of decentralized energy trading. These plots together endorse the use of adaptive pricing mechanisms to improve fairness, efficiency, and customer advantage in P2P energy marketplaces.

**Fig. 12:**
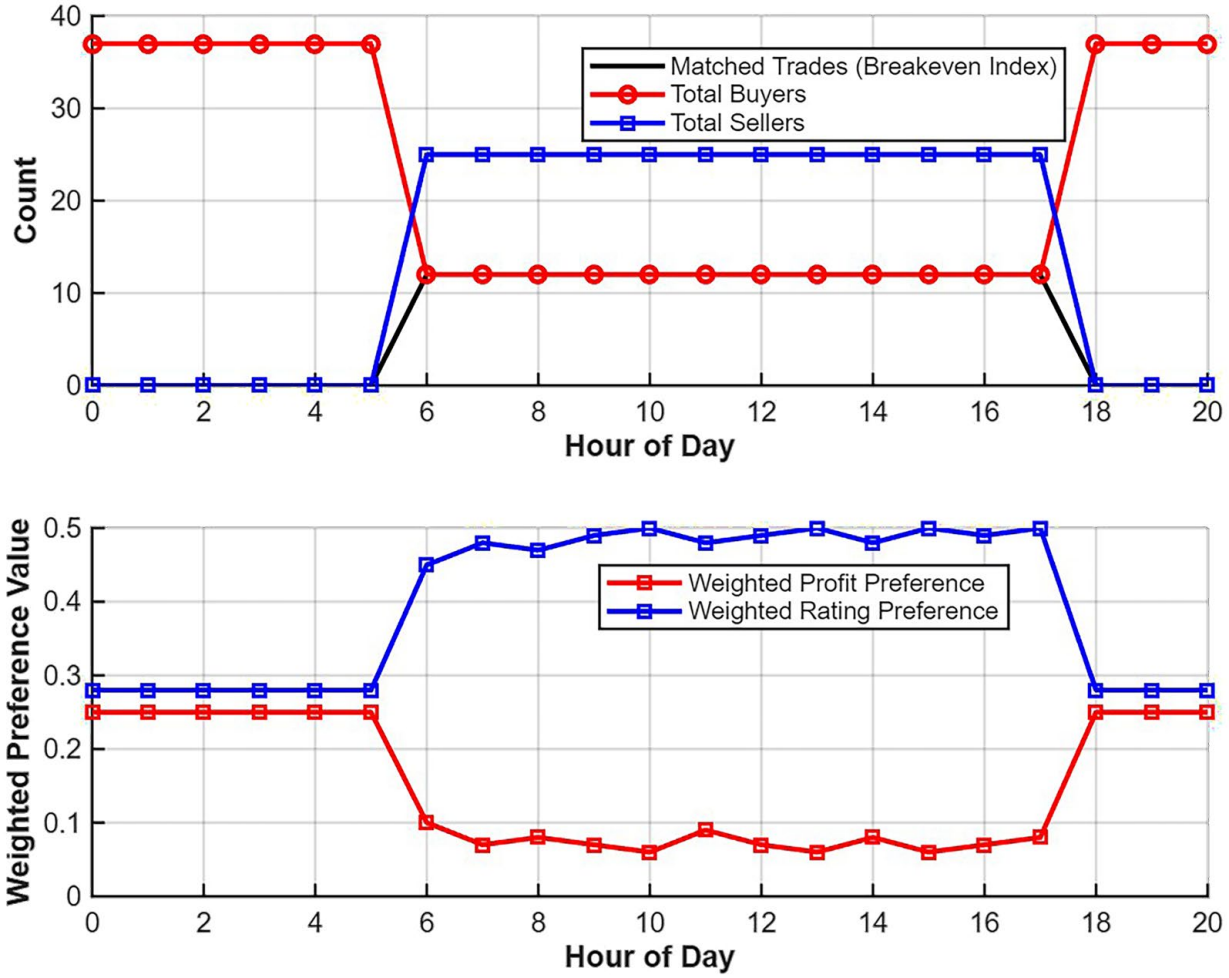
Hourly market variations and user response patterns in P2P energy trades.

Figure 12 elucidates market dynamics and user inclinations throughout a day in a peer-to-peer (P2P) energy trading system. Subplot (a) illustrates the trends in the total number of buyers, total sellers, and matched deals (breakeven index) for each hour. Initially, from midnight until early AM, there is a significant in flow of buyers but an absence of sellers, leading to no completed deals. Between 6 AM and At 6 PM, the presence of buyers and sellers is somewhat balanced, resulting in a consistent volume of matched deals, peaking at about 20 to 25. After 6 PM, sellers diminish to none while buyers increase, resulting in the cessation of trading activity. Subplot (b) depicts the fluctuating prioritization between profit preference and rating preference among participants. Throughout the busy trading hours (6 AM to 6 PM), the preference value for ratings increases, indicating that players are increasingly predisposed to select reliable or highly rated trading partners above mere profit. Conversely, the demand for profit diminishes throughout this time, indicating a transition in trading behavior towards dependability. These patterns illustrate the evolution of trade activity and decision-making preferences throughout the day, demonstrating a balance between market accessibility and participant values in decentralized energy markets.

#### Analysis of results across multiple auction-based mechanisms

**Fig. 13:**
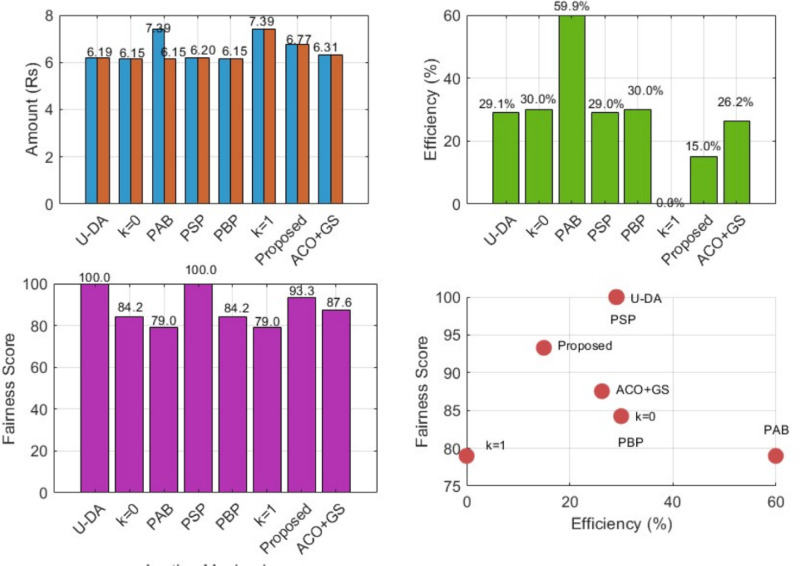
Comparision with different auction mechanisms.

Figure 13 presents a comparative analysis of multiple auction mechanisms for peer-to-peer (P2P) energy trading, utilizing real-world input data from four sellers (P1–P4) and six buyers (C5–C10). Sellers offered energy ranging from 0.38 to 0.88 kWh at rates between 3.07 ₹/kWh and 5.01₹/kWh per kWh, while buyers demanded between 0.34 and 0.44 kWh at bid prices ranging from 2.42 ₹/kWh to 5.64 ₹/kWh . The proposed Ant Colony Optimization (ACO)-based multiple-k double auction yielded a total trade value of 15.76 ₹/kWh, out performing conventional Uniform Double Auction (U-DA, 14.26 ₹/kWh) and fixed-k strategies at k = 0 ( 14.16 ₹/kWh) and k = 0.5 ( 15.58 ₹/kWh). Although Pay-As-Bid and k = 1 strategies achieved higher trade values ( 17.01₹/kWh), they potentially suffer from issues related to market inefficiencies and imbalanced buyer-seller preference matching, which may impact fairness and reliability in real-world deployment. The suggested ACO-based approach equilibrates profitability and stability by integrating factors such as ratings and profits into the matching process, there by attaining near-optimal results with enhanced fairness and efficiency. This substantiates its preeminence in a peer-to-peer energy trading context relative to conventional and fixed-parameter systems.

## Conclusion

This work presents a full market architecture for decentralized peer-to-peer (P2P) energy trading, including a multi-k double auction mechanism with constraint-aware Gale-Shapley stable matching and metaheuristic optimization. The adjustable parameter , denoting a weighted incorporation of buyer and seller price inclinations, was dynamically refined using Ant Colony Optimization (ACO).The simulation findings from the IEEE 13 and IEEE 37-node residential feeder indicated that the suggested framework improves social welfare, energy transaction efficiency, and justice, surpassing fixed-k techniques for price equity and participant satisfaction, Notwithstanding its encouraging outcomes, the model exhibits constraints in scalability and real-time responsiveness, particularly with elevated prosumer engagement. Furthermore, the methodology presupposes predictable behaviour and precise input predictions. We recommend the use of machine learning (ML) methodologies for conducting reliability study of the trading system in future research. Machine learning models may be trained on historical market and operational data to forecast system stability, identify aberrant trading patterns, and evaluate the probability of transaction failures under unknown situations. Integrating predictive reliability analytics with the suggested market framework may improve decision-making, guaranteeing resilient, secure, and adaptable energy trading in fluctuating smart grid settings.

## Data Availability

The dataset used in this study is available from the corresponding author upon reasonable request for research purposes.

## Code availability

The MATLAB simulation code implementing the hybrid market design for decentralized energy trading, including the Multi-k Double Auction and Enhanced Stable Matching framework, is publicly available in the Zenodo repository at: https://doi.org/10.5281/zenodo.18975009.

## Materials availability

All materials used in this study are commercially available.
